# Reviewer acknowledgment

**DOI:** 10.1186/1742-6405-10-6

**Published:** 2013-03-13

**Authors:** Kailash Gupta

**Affiliations:** 1 Editor-in-Chief AIDS Research and Therapy

## Abstract

**Contributing reviewers:**

A peer-reviewed journal would not survive without the generous time and insightful comments of the reviewers whose efforts often go unrecognized. *AIDS Research and Therapy* has been blessed by the support of highly qualified peer reviewers and would like to show its appreciation by thanking the following for their assistance with review of manuscripts for the journal in 2012.

## 

Upasna Agarwal

India

Kathleen Akgun

United States of America

Ramesh Akkina

United States of America

Mehmet Numan Alp

Turkey

Claudia Alteri

Italy

Jintanat Ananworanich

Thailand

Usharani Anaparthy

India

Thanomsak Anekthananon

Thailand

Spinello Antinori

Italy

Sasha Appleton

United Kingdom

Vasilios Athyros

Greece

Melanie Bacon

United States of America

Giorgio Barbarini

Italy

Kim Begley

Australia

Edward Berger

United States of America

Luigi Buonaguro

Italy

Vera Luiza Capelozzi

Brazil

Catherine Cherry

Australia

Ploenchan Chetchotisakd

Timor-Leste

Janice Clements

United States of America

Anali Conesa-Botella

Belgium

Luis Cubano

Puerto Rico

Antonella D'arminio Monforte

Italy

Anand Date

United States of America

Cynthia Derdeyn

United States of America

Chris Destache

United States of America

Pere Domingo

Svalbard And Jan Mayen

Andrew Edmonds

United States of America

Roberto Esposito

Italy

Lydia Feinstein

United States of America

Caroline Foster

United Kingdom

Joel Gallant

United States of America

Jose M Gatell

Spain

Patricia George

United States of America

Nicola Gianotti

Italy

Magnus Gisslen

Sweden

Frances Gotch

United Kingdom

Michael Gottesman

United States of America

Nathan Hansen

United States of America

Dennis Hartigan-O'connor

United States of America

Bernard Hirschel

Switzerland

Tara Horvath

United States of America

Wei Huang

United States of America

Robin Huebner

United States of America

Patrick Jean-Philippe

United States of America

Angela Kaida

Canada

Peter Kamerman

South Africa

Steve Kanters

Canada

Stephen Kerr

Australia

Arifa Khan

United States of America

Kristina W. Kintziger

United States of America

Eugene Kroon

Thailand

Adarsh Kumar

United States of America

Olivier Lambotte

France

Jairam Lingappa

United States of America

Carmen Logie

Canada

Eskindir Loha

Ethiopia

Lucia Lopalco

Italy

Vladimir V. Lukashov

Netherlands

Lut Lynen

Belgium

Frank Maldarelli

United States of America

Weerawat Manosuthi

Thailand

David Margolis

United States of America

Norman Markowitz

United States of America

Gary Marks

United States of America

Justin Mcarthur

United States of America

Ana-Claire Meyer

United States of America

Patrick Miailhes

France

Joann Mican

United States of America

Ethan Moitra

United States of America

Eve Mokotoff

United States of America

Andrew Mujugira

Uganda

Robert L. Murphy

United States of America

Satoshi Nagasaka

Japan

Kenrad Nelson

United States of America

Nancy Nguyen

United States of America

Lena Nilsson Schönnesson

Sweden

Ann O'leary

United States of America

Antonio Pacheco

Brazil

Kovit Pattanapanyasat

Thailand

Venkateswara Pavuluri

United States of America

Santiago Perez Cachafeiro

Spain

Maya Petersen

United States of America

Nittaya Phanuphak

Thailand

Vinayaka Prasad

United States of America

Garrett Prestage

Australia

Patricia Price

Australia

Lynn Ramirez

United States of America

Lokinendi Rao

United States of America

Peter Rebeiro

United States of America

Andrew Revell

United Kingdom

Doug Richman

United States of America

Giuliano Rizzardini

Italy

Robert Robey

United States of America

Jessica Robinson-Papp

United States of America

Maria Roura

Spain

Barbara Schmidt

Germany

Anjali Sharma

United States of America

Gerald Sharp

United States of America

Margie Skeer

United States of America

Daniel Skiest

United States of America

Greg Spear

United States of America

Stephen Spector

United States of America

Narasimhachar Srinivasakumar

United States of America

Jack Stapleton

United States of America

Phyllis Tien

United States of America

Vivian Towe

United States of America

Avnish Tripathi

United States of America

Marius Troseid

Norway

Drago Turcinov

Croatia

Marc van der Valk

Netherlands

Mark Wainberg

Canada

James Walkup

United States of America

Marie Wang

United States of America

Johanna Weiss

Germany

William Weiss

United States of America

Samantha Westrop

United Kingdom

Lori Wiener

United States of America

Ed Wilkins

United Kingdom

Paige Williams

United States of America

Jeffrey Wilusz

United States of America

Benjamin Young

United States of America

